# Comparative transcriptome analysis reveals the involvement of an MYB transcriptional activator, *SmMYB108*, in anther dehiscence in eggplant

**DOI:** 10.3389/fpls.2023.1164467

**Published:** 2023-07-11

**Authors:** Ruolin Hu, Jiali Wang, Huiqing Yang, Dayong Wei, Qinglin Tang, Yang Yang, Shibing Tian, Zhimin Wang

**Affiliations:** ^1^College of Horticulture and Landscape Architecture, Southwest University, Chongqing, China; ^2^Chongqing Key Laboratory of Olericulture, Chongqing, China; ^3^The Institute of Vegetables and Flowers, Chongqing Academy of Agricultural Sciences, Chongqing, China

**Keywords:** eggplant, MYB transcription factor, anther dehiscence, regulation of gene expression, RNA-seq

## Abstract

Male sterility is a highly attractive agronomic trait as it effectively prevents self-fertilization and facilitates the production of high-quality hybrid seeds in plants. Timely release of mature pollen following anther dehiscence is essential for stamen development in flowering plants. Although several theories have been proposed regarding this, the specific mechanism of anther development in eggplant remains elusive. In this study, we selected an R2R3-MYB transcription factor gene, *SmMYB108*, that encodes a protein localized primarily in the nucleus by comparing the transcriptomics of different floral bud developmental stages of the eggplant fertile line, F142. Quantitative reverse transcription polymerase chain reaction revealed that *SmMYB108* was preferentially expressed in flowers, and its expression increased significantly on the day of flowering. Overexpression of *SmMYB108* in tobacco caused anther dehiscence. In addition, we found that *SmMYB108* primarily functions as a transcriptional activator *via* C-terminal activation (amino acid 262–317). Yeast one-hybrid and dual-luciferase reporter assays revealed that genes (*SmMYB21*, *SmARF6*, and *SmARF8*) related to anther development targeted the *SmMYB108* promoter. Overall, our results provide insights into the molecular mechanisms involved in the regulation of anther development by *SmMYB108*.

## Highlights

• Excavating the differentially expressed gene *SmMYB108* of eggplant fertile line F142 in anther different developmental stages.

• Elucidating that *SmMYB108* functioned as a transcriptional activator to regulate anther development.

• Finding some genes related to anther development directly targeted *SmMYB108* promoter.

• The research results are of great significance to understand the molecular mechanism of *SmMYB108* regulating anther development.

## Introduction

Eggplant (*Solanum melongena*), an important vegetable crop, cultivated in tropical, subtropical, and temperate regions, exhibits heterosis and is mainly produced by hybrids. The use of a strict pollination control system is a prerequisite to avoid self-fertilization in plants (Kempe and Gils, 2011). Male sterility is generally used to produce hybrid seeds that can effectively avoid self-fertilization and solve the difficulties of traditional seed production. Molecular mechanisms of male infertility have been reported in previous studies (Ma et al., 2015; Singh et al., 2015). RNA-sequencing (RNA-seq) technology helps in understanding the gene expression patterns and inferring the candidate genes related to male infertility. Many studies have conducted RNA-seq analyses of the male sterile floral organs of tomatoes (Jeong et al., 2014), cotton (Wei et al., 2013), peppers (Liu et al., 2013), *Brassica napus* (Yan et al., 2013), and watermelons (Rhee et al., 2015).

Stamen is the male reproductive organ of flowering plants, which consists of an anther, a space for pollen development, and a filament that provides the anther with structural support and nutrients. Development of anther, an important component of stamens, is mainly divided into two stages. In the first stage, the initial stamen primordium forms a complete pollen sac wall after tissue specialization and meiosis, which is followed by the formation of the outer epidermis, endothecium, middle layer, and tapetum from the outside to the inside. Tetrad structure of the microspores is located at the center of the anther chamber. In the second stage, the anther continues to expand, filament elongates, microspores develop into pollen grains from the tetrad structure, pollens sac cracks and releases pollens, and pollination and fertilization are completed, after which, the anther tissue gradually ages and degenerates (Goldberg et al., 1993; Wilson et al., 2011).

An anther dehisces in a timely manner to release mature pollen for complete pollination and fertilization. Transcription factors (TFs) containing the MYB domain in plants are known as MYB TFs. MYB domain consists of one to four amino acid (aa) sequences and forms a helix-turn-helix motif (Dubos et al., 2010). On the basis of the number of adjacent repeats, MYB proteins are divided into four categories: 1R-MYB (MYB-related), 2R-MYB (R2R3-MYB), 3R-MYB (R1R2R3-MYB), and 4R-MYB (Katiyar et al., 2012). Among them, R2R3-MYB TFs are one of the most abundant gene families in plants (Stracke et al., 2001) that are involved in plant cell development and morphogenesis, biological and non-biological stress responses, hormone signal transduction, and secondary metabolism regulation (Wang et al., 2016; Kocábek et al., 2018; Guan et al., 2019).

Secondary thickening of the inner wall of the anther chamber is essential for anther dehiscence (Zhao and Dong, 2015). *AtMYB26* is vital for secondary thickening, directly inducing the expression of *AtNST1* and *AtNST2* in the anthers. Both *myb26* and *nst1 nst2* mutants failed to exhibit anther dehiscence (Yang et al., 2017). Previous studies have reported that *AtMYB103*, *AtMYB83*, and *AtMYB46* are direct targets of *AtNST1* and *AtNST2*, regulating secondary wall biosynthesis to affect anther dehiscence and pollen development. Another study reported that *AtMYB83* acts redundantly with *AtMYB46* as a key molecular in the regulating of secondary wall biosynthesis (Zhong et al., 2008; McCarthy et al., 2009; Zhong et al., 2007). Li et al. (2019) revealed that *RUS*4 affects the synthesis of the secondary wall of the inner wall of the drug chamber by indirectly activating the expression of the TFs, *AtMYB103* and *AtMYB85* (Li et al., 2019). Because of the defects in filament elongation, anther dehiscence, and pollen viability, *myb21 myb24* double-mutant plants are male sterile. R2R3-MYB TFs, *MYB21 a*nd *MYB24*, are essential for filament elongation, anther dehiscence, and pollen maturation, jasmonic acid (JA) regulation of male fertility in *Arabidopsis thaliana* (Song et al., 2011). Overexpression of *GhMYB24* in Arabidopsis severely decreases the fertility by inhibiting filament elongation and anther dehiscence and decreasing the pollen viability. *atmyb21 atmyb24* double-mutant plants have been reported to be severely male sterile, and the expression of *GhMYB24* in the *atmyb21 atmyb24* double-mutant has been reported to partially rescued the sterile phenotype (Li et al., 2013). Several studies have investigated the role of the TF, *MYB108*, in anther development. *AtMYB108* and *AtMYB24* have been reported to coordinate the JA pathway during anther dehiscence (Mandaokar and Browse, 2009). Sun et al. reported that a JA-induced R2R3-MYB TF, *CaMYB108*, is involved in the regulation of capsaicinoids, cap biosynthesis, and stamen development. Silencing of *CaMYB108* has been reported to delay anther dehiscence and decrease the pollen viability (Sun et al., 2019). *arf1*7 mutant anther is indehiscent, and the auxin response factor, *ARF17*, can directly bind to the *AtMYB108* promoter and induce its expression in *arf17* mutant to rescue its anther dehiscence phenotype (Xu et al., 2019).

Molecular mechanism by which TF *MYB108* regulates the development of anthers remains poorly understood. In this study, we identified differentially expressed gene (DEG), *SmMYB108*, in eggplant, whose expression was highest on the day of flowering (FD). We verified that this TF was involved in stamen development. In addition, we found that proteins related to anther development (SmMYB21, SmARF6, and SmARF8) directly bound to the promoter of *SmMYB108*. Our findings indicate that *SmMYB108* plays a central role in anther development.

## Results

### Identification of DEGs

**Figure 1:**
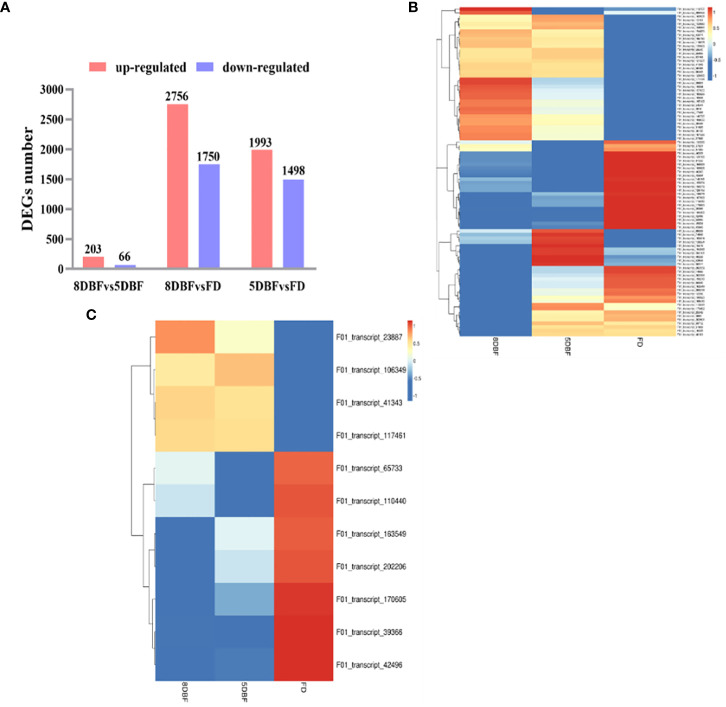
Analysis of DEGs between the eggplant fertile line F142 in different stages of flower buds. **(A)** The total number of upregulated and downregulated DEGs. **(B)** Heat map of differentially expressed transcription factors. **(C)** Heat map analysis of 11 common differentially expressed genes.

We determined the mRNA (messenger Ribonucleic Acid) expression levels in the floral buds of the eggplant fertile line, F142, at different developmental stages. The FPKM (fragments per kilobase of exon per million mapped fragments) method was used to identify the DEGs. In total, 5,549 DEGs were identified *via* pairwise comparisons. Comparison of 8 days before flowering (DBF) and 5 DBF revealed the lowest number, 269 DEGs, of which 203 were upregulated and 66 were downregulated. The maximum number of DEGs was between 8 DBF and the day of flowering (FD), including 2,756 upregulated and 1,750 downregulated genes (**Figure 1A**). Notably, 2,552 common DEGs (1,739 upregulated and 816 downregulated) were shared between the 8 DBF and FD and between 5 DBF and FD groups (**Supplementary Figures S1A–C**).

TFs play an important role in anther development. Eighty-nine TFs were identified among all DEGs, which could be classified into 29 families. The important families were bHLH (basic helix-loop-helix) (19.10%), MYB(v-myb avian myeloblastosis viral oncogene homolog)-related (13.48%), AP2 (APETALA2) (11.24%), NAC (NAM、ATAF1/2、CUC1/2) (1.12%), WRKY (7.87%), TCP ((TEOSINTE BRANCHED1/CYCLOIDEA/PROLIFERATING CELL FACTORS) (7.87%), and the MADS (2.25%) families (**Supplementary Figure S2A**). On the basis of the similarity in gene expression, hierarchical clustering was performed using the 89 differentially expressed TFs in samples (**Figure 1B**; **Supplementary Table 2**). Hierarchical clustering of the gene expression profiles in the three stages revealed different expression patterns in these samples, providing an overall understanding of the changes in gene expression. Forty-two MYB TFs were identified among 89 TFs, of which 31 were upregulated and 11 were downregulated. Moreover, 11 common MYB TFs between 8 DBF and FD and between 5 DBF and FD were identified, and the expression pattern was visualized using a heat map (**Supplementary Figure S2B**; **Supplementary Table 3**). Interestingly, we found that the expression levels of gene F01_transcript_42496 (referred as *SmMYB108* in this study) were significantly higher in FD than that in 8 and 5 DBF (**Figure 1C**). These results suggest the involvement of an extremely intricate transcriptional network in anther development.

### *SmMYB108* is highly expressed during the flowering period

**Figure 2:**
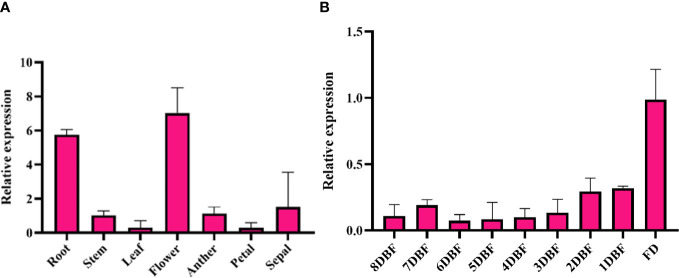
Expression analysis of *SmMYB108*. **(A)** Expression of *SmMYB108* in different tissues. **(B)** Expression of *SmMYB108* in different developmental stages of flower buds.

To verify the RNA-seq data, quantitative reverse transcription polymerase chain reaction (qRT-PCR) was conducted on nine randomly selected genes, and the results confirmed the accuracy of transcriptome analysis (**Supplementary Figure S3**). We found that *SmMYB108* was predominantly expressed in the flowers, followed by roots, but its transcript levels were low in the stems, leaves, anthers, petals, and sepals through qRT-PCR (**Figure 2A**). Moreover, we quantitatively analyzed the expression levels of *SmMYB108* at different developmental stages. Within the floral buds, *SmMYB108* transcript level was stable from 8 to 3 DBF, increased slightly 2 DBF, and reached the maximum level at flowering (**Figure 2B**). Taken together, our results indicate *SmMYB108* as a candidate regulator of anther development. Moreover, *SmMYB108* may have roles other than anther development regulation in plants.

**Figure 3:**
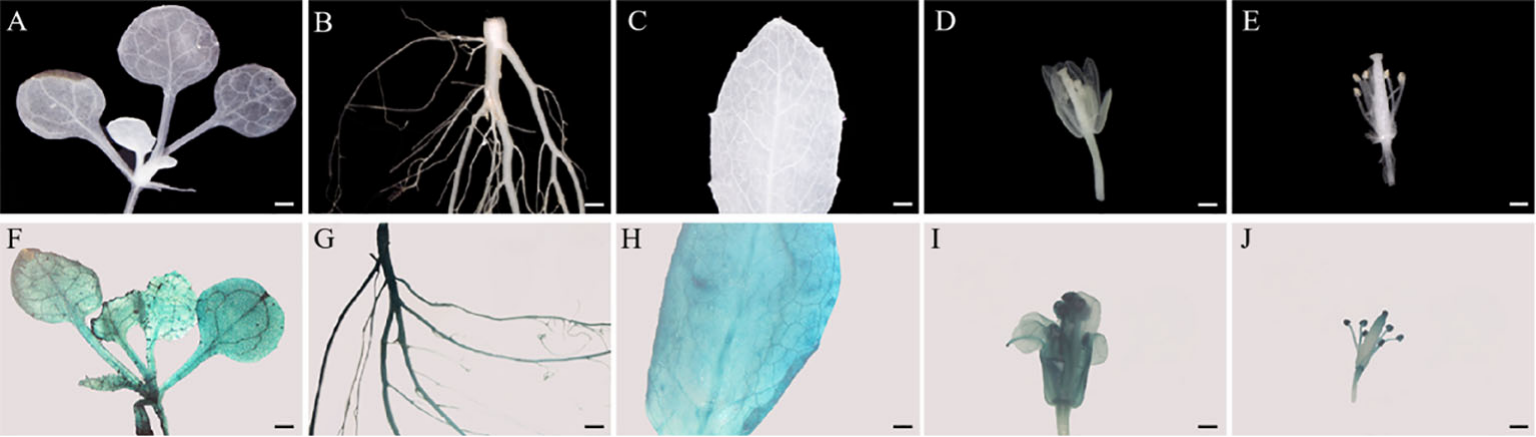
Tissue-specific expression analysis of *SmMYB108* in *prSmMYB108:GUS* transgenic plants. **(A–E)** Seedling, root, leaf, mature flower, and anther (from left to right) were from wild type. **(F–J)** Gus-stained seedling, root, leaf, mature flower, and anther (from left to right) were from *prSmMYB108:GUS* transgenic plants. Bar, 500 µm.

To visualize the detailed expression patterns of *SmMYB108*, its promoter was cloned approximately 2-kb 5′ upstream of the sequence included in *gSmMYB108* in this study. We generated *prSmMYB108:GUS Arabidopsis* transgenic lines expressing GUS (Β-glucuronidase) fused with the promoter sequence of *SmMYB108* to detect *SmMYB108* expression patterns. Consistent with the gene expression pattern, blue staining was high in the roots, flowers, and leaves of mature plants (**Figures 3B–I**) and mainly observed in the mature anthers in flowers (**Figures 3E, J**). GUS staining was observed in all organs in approximately 2-week-old seedlings (**Figures 3A, F**). These findings highlight the potential role of *SmMYB108* in anther development.

### *SmMYB108* encodes a nucleus-localized R2R3 MYB TF

**Figure 4:**
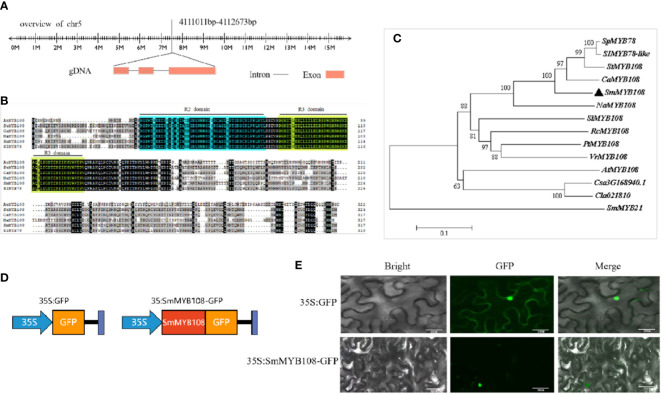
Phylogenetic and subcellular analyses of SmMYB108. **(A)** The genomic sequence of *SmMYB108* that is mapped to eggplant genome. **(B)** Sequence analysis of *SmMYB108*. To compare the MYB sequences among different plant species, *SmMYB108* was used as a bait gene and searched in National Center for Biotechnology Information (NCBI) database for highly similar sequences MYB TFs in other species. **(C)** A phylogenetic tree showing aa sequence similarities among *SmMYB108* and closely related MYB TFs in other species. **(D, E)** Subcellular localization of 35S:SmMYB108-GFP fusion protein in Tobacco epidermal cells. 35S::GFP served as a control. Bar, 50 µm.

Full-length coding sequence (CDS) of *SmMYB108* was isolated from the eggplant fertile line F142, and its open reading frame was 954 bp, encoding a protein of 317 amino acids, located on chromosome 5 (**Figure 4A**). Aa sequence analysis revealed that *SmMYB108* contains two motif DNA-binding domains (BDs) (R2-R3 MYB type) next to the N-terminus (**Figure 4B**). We retrieved *SmMYB108* homologs from *Solanum lycopersicum*, *S*. *tuberosum*, *Nicotiana attenuata*, *Capsicum annuum*, and *A*. *thaliana*. A phylogenetic tree was constructed to identify the orthologs of these genes. We found that the nearest MYB TFs from *StMYB108* (*S*. *tuberosum*), *CaMYB108* (*C*. *annuum*), *SlMYB78* (*S*. *lycopersicum*), and *NaMYB108* (*N*. *attenuata*), shared sequence identity > 90% (**Figure 4C**). *AtMYB108*, which showed only 60% aa sequence identity, was the MYB TF most closely related to *A*. *thaliana*. Interestingly, both *CaMYB108* and *AtMYB108* were induced by JA to participate in anther development (Mandaokar and Browse, 2009; Sun et al., 2019), suggesting that *SmMYB108* may also play a role in stamen development.

TFs regulate transcription depending on their localization in the nucleus. To determine the subcellular localization of *SmMYB108*, the CDS of *SmMYB108* was fused in frame with the green fluorescence protein (GFP) to generate a *35Spro::GFP-SmMYB108* fusion protein (**Figure 4D**). *35Spro::GFP-SmMYB108* was transiently expressed in the nucleus of *N*. *benthamiana* leaf epidermal cells. GFP-SmMYB108 was observed exclusively in the nucleus, whereas free GFP (35S::GFP) was observed in both the cytoplasm and nucleus of epidermal cells (**Figure 4E**), suggesting that *SmMYB108* is localized in the nucleus.

### Overexpression of *SmMYB108* promotes stamen development

**Figure 5:**
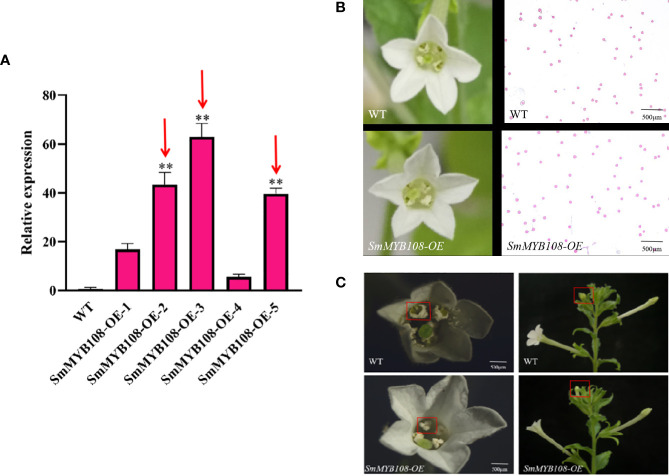
Morphological characterization of *SmMYB108-OE* plants floral organ. **(A)** RT-qPCR analysis of SmMYB108 transcripts present in wild-type (WT) and *SmMYB108-OE* lines. Error bars, 6 SEM (n = 3). Three lines were selected for further research (red arrows). **(B)** Morphological features of flowers in the same period in wild type and *SmMYB108-OE* lines. **(C)** The degree of antherin the same period in wild type and SmMYB108-OE lines.

To further study the function of *SmMYB108*, we constructed an overexpression vector used for the *Agrobacterium*-mediated transformation of tobacco. We successfully developed 14 transgenic tobacco plants overexpressing *SmMYB108*. In T_0_ plants, *SmMYB108-OE-2*, *SmMYB108-OE-*3, and *SmMYB108-OE-5* were selected for further analysis (**Figure 5A**). Floral organ morphology and pollen vitality were not significantly different between the wild-type and *SmMYB108-OE* lines as they exhibited normal anther dehiscence and released viable pollen (**Figure 5B**). However, the anthers of transgenic tobacco were completely dehiscent and covered by pollen, whereas those of wild-type tobacco were partially dehiscent and covered by a small amount of pollen. These results revealed that the degree of anther dehiscence in transgenic tobacco was greater than that in wild-type tabacco. Moreover, flower buds immediately at the top of tobacco developed earlier than those in transgenic tobacco and wild-type plants. Taken together, these results suggest that *SmMYB108* overexpression promotes the early dehiscence of anthers (**Figure 5C**).

### *SmMYB108* functions as a transcriptional activator

**Figure 6:**
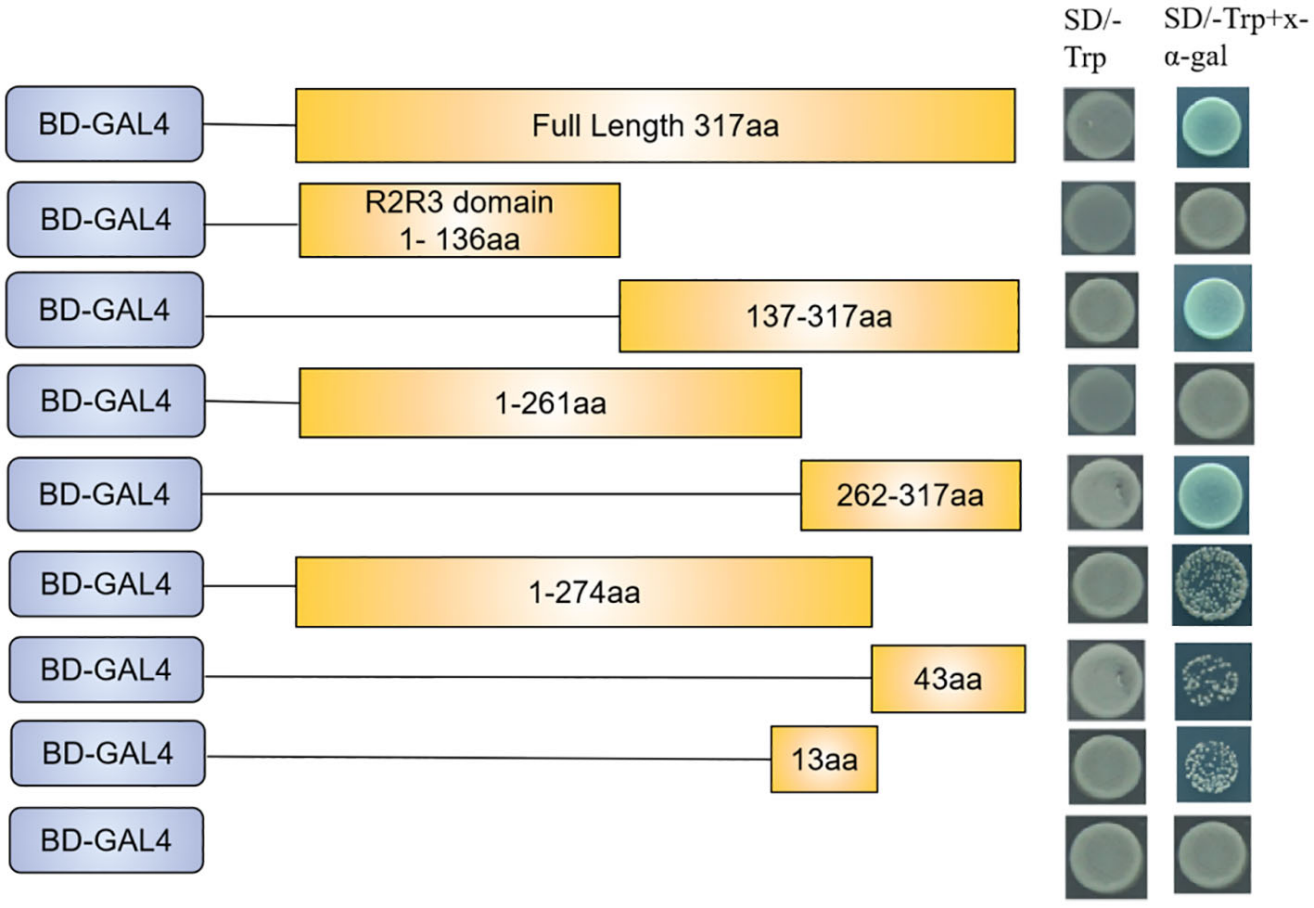
*SmMYB108* is a transcription activator. Activation analysis of *SmMYB108* in yeast. Full-length and truncated SmMYB108 was used for activation analysis. The number shown on the right indicates the protein region used for activation analysis. Auxotroph plates of SD/–Trp(left) and SD/–Trp–x-α-gal(right) showing transcriptional activation of protein.

*SmMYB108* contains an N-terminal R2R3 repeat domain, responsible for DNA binding, and a C-terminal domain. Transcriptional activation domain of the R2-R3 type MYB TF, which encodes the aa, was located at the C-terminus, as previously reported (Song et al., 2011). We performed a transactivation activity assay in yeast to test the transcriptional activation activity of *SmMYB108*. On the basis of the presence of conserved domains, full-length and a series of truncations were fused in-frame with the GAL4 DNA-binding domain (BD) in the pGBKT7 (BD) vector, and the constructs were transformed into the yeast two-hybrid (Y2H) gold yeast strain. Yeast carrying BDSmMYB108 grew and turned blue on SD/−Trp selective medium along with X-α-gal, indicating that TF *SmMYB108* acts as a transcriptional activator (**Figure 6**). To determine the specific domain influencing the activation activity of *SmMYB108*, the constructed truncated SmMYB108 was transformed into the Y2H gold yeast strain (**Figure 6**). The results showed reduced transcriptional activation of SmMYB108 (275–317 aa) and SmMYB108 (262–274 aa), indicating destruction of the self-activating domain. Therefore, the C-terminus (262–317 aa) was essential for the activation activity of *SmMYB108* (**Figure 6**).

### Anther development-related genes directly target *SmMYB108*

**Figure 7:**
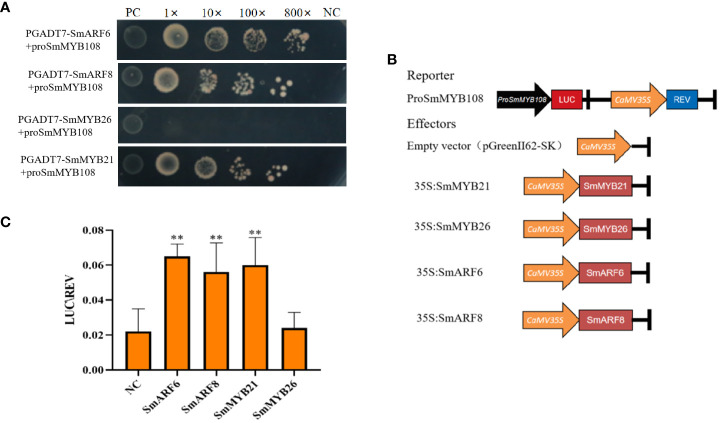
Anther development-related genes targets the promoters of *SmMYB108*. **(A)** Y1H analysis of SmMYB21, SmARF6, and SmARF8 associated with the promoters of SmMYB108. **(B)** ProSmMYB108, empty vector (pGreenII62-SK), 35S:SmMYB21, 35S:SmMYB26, 35S:SmARF6, and 35S:SmARF8 were infected into *N. benthamiana* cells. **(C)** Dual-luciferase reporter assay of SmMYB21, SmARF6, and SmARF8 activated the transcription of SmMYB108 promoters. Values are the mean ± SD of six replicates. One-way ANOVA was used to identify significant differences (**p < 0.01).

*AtMYB21*, *AtMYB26*, *AtARF6*, and *AtARF8* participated in anther development in *A*. *thaliana*, as previously reported (Song et al., 2011; Yang et al., 2017; Nagpal et al., 2005). To determine the direct target genes of *SmMYB108*, we selected *SmMYB21*, *SmMYB26*, *SmARF6*, and *SmARF8* as candidate target genes for screening *via* Y1H assay. *SmMYB108* promoters were ligated into the pAbAi vector to construct bait vectors (pAbAi-ProSmMYB108). *SmMYB21*, *SmMYB26*, *SmARF6*, and *SmARF8* were ligated into the pGADT7 vector to construct a prey vector. Subsequently, bait and prey vectors were transformed into the Y1H gold yeast strain. As shown in **Figure 7A**, the negative control (pGADT7 + ProSmMYB108) and transformant (pGADT7-SmMYB26 + ProSmMYB108) did not grow well on the medium containing aureobasidin A (AbA; 600 ng ml^−1^). However, the positive control (p53 + Prop53) and transformants (pGADT7-SmMYB21 + ProSmMYB108, pGADT7-SmARF6 + ProSmMYB108, and pGADT7-SmARF8 + ProSmMYB108) grew well on the medium containing AbA. These findings indicate that *SmMYB21*, *SmARF6*, and *SmARF8* are associated with the *SmMYB108* promoters *in vitro*.

Next, we used a dual-luciferase (LUC) reporter assay to confirm whether *SmMYB108* promoter transcription could be activated by *SmMYB21*, *SmMYB26*, *SmARF6*, and *SmARF8 in vivo*. *SmMYB108* promoter fragment was fused to a firefly LUC reporter sequence and co-transformed into tobacco leaves either with 35S:SmMYB21, 35S:SmMYB26, 35S:SmARF6, and 35S:SmARF8 effector constructs or with an empty vector before determining the relative LUC activity using the LUC/Renilla (REN) ratio (**Figure 7B**). We found that *SmMYB21*, *SmARF6*, and *SmARF8* activated the transcription of *SmMYB108* promoter in a gene-dependent manner. *SmMYB108* promoter transcription was strongly activated by *SmMYB21*, *SmARF6*, and *SmARF8* in *N. benthamiana* leaves. However, no difference was observed between *SmMYB108* promoter-driven and empty vector LUC activity (**Figure 7C**). Taken together, these results indicate that *SmMYB21*, *SmARF6*, and *SmARF8* are the transcriptional activators of *SmMYB108.*

## Discussion

RNA-seq is used to determine the gene expression patterns and analyze DEGs responsible for genetic variation. Transcriptome analysis is often used to identify the DEGs involved in male sterility in various species (Jeong et al., 2014; Wang et al., 2021; Yuan et al., 2021). In this study, we conducted RNA-seq to determine the gene expression changes at different developmental stages (8 DBF, 5 DBF, and FD) in eggplant floral buds. Overall, 5,549 genes were identified *via* pairwise comparisons (**Figure 1A**). The anther is not only an important part of the stamens but also the place of pollen development. Normal dehiscence of anthers and timely release of pollen are essential for the completion of pollination and fertilization in plants (Goldberg et al., 1993; Wilson et al., 2011). Various TFs, including MYB TFs, play an important role in anther development (Veenstra and Wolffe, 2001). We identified 11 common MYB TFs in 8 DBF vs. FD and 5 DBF vs. FD groups. The expression of *SmMYB108* (F01_transcript_42496) was significantly increase on the FD (**Figure 1C**). Nine genes among all DEGs were randomly selected, and their expression levels were of consistent in both platforms (**Supplementary Figure S3**). qRT-PCR analysis revealed that the expression of *SmMYB108* was highest on the FD (**Figure 2**). *pSmMYB108:GUS Arabidopsis* transgenic lines also exhibited consistent gene expression patterns (**Figure 3**).

N-terminal of MYB TFs has a conserved MYB domain, which is divided into four types: 4R-MYB, 2R-MYB (R2R3-MYB), 3R-MYB (R1R2R3-MYB), and 1R-MYB (MYB-related) (Katiyar et al., 2012). We identified a novel MYB TF, *SmMYB108*, in the nucleus that had a length of 317 aa. It encoded a R2R3-MYB protein in the N-terminus and was involved in anther development. (**Figure 4E**). Sequence analysis of *SmMYB108* and its orthologs revealed that *CaMYB108*, *StMYB108*, *SlMYB78*, and *NaMYB108* shared a fairly high sequence identity with *SmMYB108* (>90% aa sequence identity), whereas *AtMYB108*, which is most closely related to *SmMYB108*, shared only 60% sequence identify. JA-inducible *AtMYB108* has been reported to mediate stamen and pollen maturation as well as response to pathogens. Silencing the JA-inducible *CaMYB108* resulted in delayed anther dehiscence, decreased pollen viability, and promoted the synthesis of capsaicin. In our study, phenotypic analysis of transgenic tobacco revealed that the overexpression of *SmMYB108* promoted anther dehiscence (**Figure 5C**). Our results indicated an intriguingly convergent evolution of *MYB108* gene function among different plant species (Mandaokar and Browse, 2009; Sun et al., 2019); however, *MYB108* gene function is known to differ among different plant species. Zhang et al. (2019) reported that *RhMYB108* participates in the interaction between ethylene and JA signals and is strongly expressed in rose petals. Silencing *RhMYB108* promoted the expression of senescence associated genes, delaying the petal senescence (Zhang et al., 2019). Both *PsnMYB108* and *MdMYB108L* mediate plant stress resistance and can improve plant salt tolerance under abiotic stress (Wang et al., 2019; Zhao et al., 2020). However, the specific roles of *SmMYB108* in different species require further investigation.

In addition to the conserved R2R3 DNA-BD, the C-terminus performs several vital functions (Dubos et al., 2010). Transcriptional activation domain of *CaMYB31* is at the C-terminus (213–234 aa) (Zhu et al., 2019). *CaMYB48* has a motif that is rich in amino acids at the C-terminus (168–191 aa) that functions as a transcriptional activation domain (Sun et al., 2020). In this study, transcriptional activation analysis of yeast showed that C-terminus (262–317 aa) was crucial for the activation activity of *SmMYB108* (**Figure 6**). Multiple transcriptional binding sites were identified in the *myb108* promoter sequence. Y1H and dual-LUC reporter assays revealed that *SmMYB21*, *SmARF6*, and *SmARF8*, but not *SmMYB26*, could activate the *SmMYB108* promoter (**Figure 7**). *AtARF6* and *AtARF8* promote anther dehiscence in floral buds (Nagpal et al., 2005). JA and auxin are important hormones in plant development and stress response, which are also involved in the regulation of anther dehiscence. *AtARF6* and *AtARF8* regulate anther dehiscence by activating *DAD1*, a key gene in JA biosynthesis, to mediate JA synthesis (Tabata et al., 2010). Moreover, *AtMYB21* mediates the JA pathway during anther dehiscence (Song et al., 2011). Whether *SmMYB108* also regulates anther development *via* the hormone pathway needs to be investigated further in future studies.

## Conclusion

In summary, we found that the promoter of *SmMYB108* directly combines with the targets genes related to anther development—*SmMYB21*, *SmARF6*, and *SmARF8*, but not *SmMYB26.* We also found that *SmMYB108* functions as a transcriptional activator to regulate anther development. Our findings provide mechanistic insights into the evolution of male sterility in plants and can aid in future investigation of the transcriptional regulatory networks involved in anther development in eggplant.

## Materials and methods

### Plant materials

F142, a fertile eggplant (*Solanum melongena*) line, was provided by the Institute of Vegetables and Flowers, Chongqing Academy of Agricultural Sciences (Chongqing, China). All plants were cultivated in a controlled chamber (16/8-h light/night photoperiod and 28/22°C light/night temperature with 60%~65% relative humidity). Floral buds were collected respectively at 8 DBF, 5 DBF, and the FD for transcriptome sequencing. Various tissues (roots, stems, leaves, anthers, sepal, petals, and flower buds at different periods) were collected for qRT-PCR. Every sample was set three biological replicates. *N. benthamiana* is kept and supplied by our laboratory. Wild-type *Arabidopsis thaliana* is provided by our laboratory.

### RNA-seq analysis

The transcriptome data of the fertile line F142 were retrieved from the previous studies (Wang et al., 2021; Yuan et al., 2021). These data included transcription data of genes from different developmental stages of floral buds. Raw reads were first filtered to obtain clean reads, which were aligned to the eggplant HQ-1315 genome using Hisat2 (version 2.1.0). Only reads with a perfect match or one mismatch were further analyzed and annotated based on the reference genome. Each gene expression level was calculated as FPKM. Differential expression analysis (three biological replicates at different developmental stages) was performed using the DESeq. Genes with an adjusted P-value <0.01, as found by DESeq, were considered as differentially expressed (Anders and Huber, 2010).

### Quantitative reverse transcription polymerase chain reaction analysis

For the extraction of total RNA from the samples, the RNAprep Pure Plant Kit polysaccharide and polyphenol total RNA extraction kit (Tianmo, China) were used. cDNA was synthesized from RNA (1 µg per sample) using the Prime Script RT Kit (Takara, China) according to the manufacturer’s instructions. Using real-time qRT-PCR validated the RNA-seq data and performed the expression of gene in different tissues. qRT-PCR primers were designed using Primer Premier 5.0 (**Supplementary Table 1**). The reactions of qRT-PCR were performed with the SYBR Green qPCR Mix (Takara, Japan) on the Bio-Rad CFX96 Touch Real Time PCR detection System (Bio-Rad Laboratories). In addition, reactions were performed three biological replicates and three technical replicates. The GAPDH (Glyceraldehyde -3-phosphate dehydrogenase) gene was used as the reference to quantify transcript levels. The 2^−△△CT^ approach was applied to calculate the relative expression levels of target genes (Livak and Schmittgen, 2001; Vandesompele et al., 2002).

### Histochemical analysis of GUS expression

A section (2 kb) of the *SmMYB108* promoter was cloned into the pGreen vector harboring the GUS reporter to construct *pSmMYB108:GUS*. Tissues for GUS staining were first infiltrated with staining solution [50 mM sodium phosphate buffer (pH 7.0), 0.5 mM potassium ferrocyanide, 0.5 mM potassium ferricyanide, and X-Gluc (0.5 mg ml^−1^)] in a vacuum chamber and, subsequently, incubated with the same solution at 37°C for 4 h.

### Sequence alignment and phylogenetic tree construction

To understand the location of the TFs on chromosome, the protein sequence of *SmMYB108* was obtained from eggplant reference genome HQ-1315. Other MYB TF sequences that showed the higher sequence identity with *SmMYB108* was retrieved from the public database National Center for Biotechnology Information (NCBI; https://www.ncbi.nlm.nih.gov/) and Sol Genomics Network (http://solgenomics.net/) and used for phylogenetic tree construction. The MYB TF sequences were aligned by CLUSTALW with the default parameters, and the phylogenetic tree was constructed by MEGA 7.0 using the neighbor-joining method with 1,000 bootstrap replications.

### Subcellular localization

The full-length *SmMYB108* cDNA was cloned from fertile line F142, which was inserted into the pCAMBIA1300 vector without the stop codon and fused with GFP-generating SmMYB108-GFP fusion proteins under the control of the CaMV35S promoter. The SmMYB108-GFP fusion proteins were introduced into Agrobacterium tumefaciens strain GV3101, which was infiltrated into tobacco leaves epidermal cells along with empty plasmids. After 48 h of inoculation, the GFP signal was visualized by confocal fluorescence microscopy (Carl Zeiss, Germany). The primers used in this study were listed in **Supplementary Table 1**.

### Transcriptional activation analysis

The ability of SmMYB108 to activate transcription was verified by a yeast transcriptional activation assay according to the manufacturer’s instructions of the Matchmaker Gold yeast two-hybrid system (Takara, China). Full-length or deleted *SmMYB108* was fused in frame with the GAL4 DNA-BD in pGBKT7 vector. The pGBKT7 empty vector that only expressed GAL4 BD was used as a negative control. Recombinant plasmids and empty vector were transformed into yeast strain Y2H Gold. Yeast cells were incubated on SD/-Trp medium at 30°C for 3 days. The positive clones were diluted in 0.9% NaCl solution, and 10 µl of each dilution was inoculated on SD/-Trp +X-α-gal medium at 30°C for 3–5 days; the clones were stained with x-α-Gal (Takara, China). The primers used in this study were listed in **Supplementary Table 1**.

### Y1H assay

The ORF (Open reading frame) of four genes (*SmMYB21*, *SmMYB26*, *SmARF6*, and *SmARF8*) were inserted into *Sma*I and *Xho*I of the pGADT7 vector to generate prey. The promoter fragments of *SmMYB108* were ligated into *Sac*I and *Sma*I of the pAbAi vector as bait. The Y1H experiment was carried out using the Matchmaker Gold yeast one-hybrid system (Takara, China) according to the manufacturer’s protocol. The primers used in this study were listed in **Supplementary Table 1**.

### Dual-LUC assay

The *SmMYB108* promoter (2,000 bp) was amplified by PCR from eggplant fertile line F142 and inserted into the *Sac*I and *BamH*I of pGreenII-0800-LUC vector to serve as the reporters. The full-length CDS of four genes (*SmMYB21*, *SmMYB26*, *SmARF6*, and *SmARF8*) was cloned into the *Sac*I and *BamH*I of pGreenII62-SK vector under the CaMV35S promoter to serve as an effector. The recombinant plasmids were transformed to the Agrobacterium tumefaciens strain GV3101 (pSoup-p19). The GV3101 harboring the *SmMYB108* reporter and the corresponding effectors were injected into 30-day-old tobacco (*Nicotiana tabacum*) leaves. After incubation for 48–72 h, the firefly LUC and REN LUC activities were measured with the Dual Luciferase Reporter Gene Assay Kit (Yisheng, Shanghai) according to the manufacturer’s introduction. The Varioskan™LUX (Thermo Fisher Scientific, USA) was used to carry out the assay. Activity was expressed as the ratio of firefly LUC activity to REN LUC activity. The primers for all constructs in this study were listed in **Supplementary Table 1**.

### Construction of a pCAMBIA-2301G-SmMYB108 vector and tobacco transformation

*SmMYB108* was generated through PCR amplification by using specific primers containing *BamH*I and *Sac*I sites. The fragments were inserted into the pCAMBIA-2301G vector (Lü et al., 2017) to form the construct pCAMBIA-2301G-SmMYB108. Agrobacterium-mediated transformation of *N. benthamiana* was then performed, as previously described (Zhao et al., 2012). The sterile tobacco leaves of 15–20 days were cut into 1-cm^2^ size into resuspension for 10 min at 25°C dark culture for 2 days and transferred to screening medium for differentiation to induce indefinite shoots; differentiated shoots were transferred to culture medium for rooting screening.

## Data availability statement

The datasets presented in this study can be found in online repositories. The names of the repository/repositories and accession number(s) can be found below: NCBI accession PRJNA941250.

## Author contributions

ZW, RH, and JW designed the research; RH and JW performed the molecular biology experiments; HY, DW, QT, YY and ST carried out the bioinformatics analysis; RH and JW analyzed the data and wrote the paper manuscript. All authors contributed to the article and approved the submitted version.
