# Supplementary material for: Comparative transcriptome analysis reveals the involvement of an MYB transcriptional activator, SmMYB108, in anther dehiscence in eggplant
Source: Front Plant Sci. 2023 Jul 11;14:1164467. doi: 10.3389/fpls.2023.1164467 (PMC10382176; doi:10.3389/fpls.2023.1164467)
Supplement: Supplementary file 1 [file DataSheet_1.pdf]

Comparative Transcriptome Analysis Reveals the Involvement of an MYB Transcriptional Activator, *SmMYB108*, in Anther Dehiscence in Eggplant

Ruolin Hu[a, b]†, Jiali Wang[a, b, †], Huiqing Yang[a, b], Dayong Wei[a, b], Qinglin Tang [a,b], Yang Yang [c], Shibing Tian [c*], Zhimin Wang [a, b, *] [a]*College of Horticulture and Landscape Architecture, Southwest University, Chongqing 400715, China*

[b] *Key Laboratory of Horticulture Science for Southern Mountainous Regions, Ministry of Education, Chongqing 400715, China*

[c] *The Institute of Vegetables and Flowers，Chongqing Academy of Agricultural Sciences，Chongqing 400055，China*

†These authors contributed equally to the work.

*Corresponding author.
E-mail addresses: minzniwang_555@163.com (Z.M. Wang), 2508436730@qq.com (S.B. Tian)

SUPPLEMENTAL FIGURES

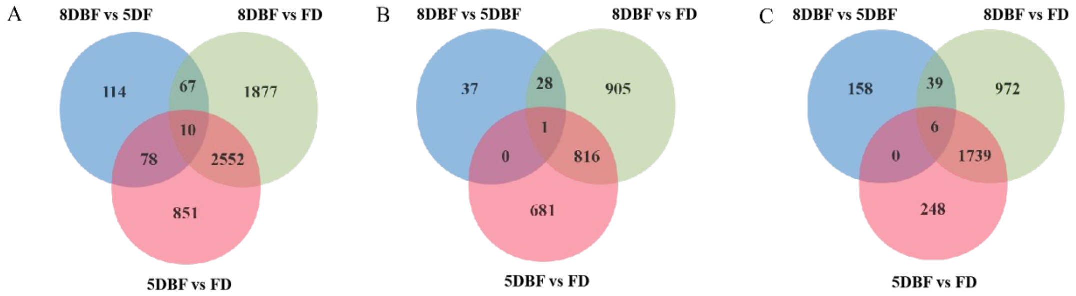

Supplementary Figure S1 Venn diagram of of DEGs between the eggplant fertile line F142 in different stages of flower buds

A: Venn diagram of all DEGs; B: Venn diagram of up-regulated genes; C: Venn diagram of down-regulated genes.

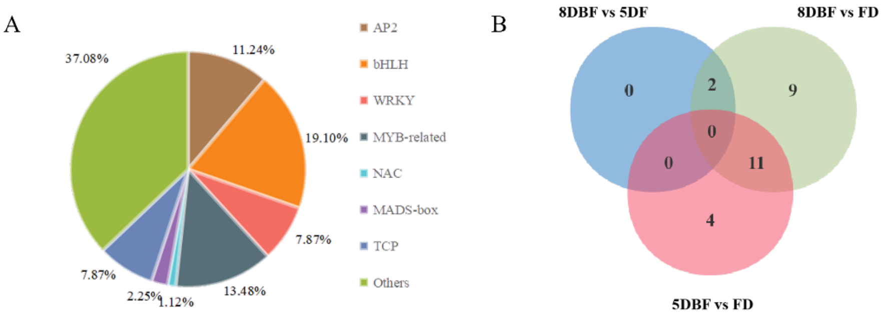

Supplementary Figure S2 Analysis of transcription factor differentially expressed gene
A: Distribution of differentially expressed transcription factors; B: Venn diagram of MYB transcription factor differentially expressed genes;

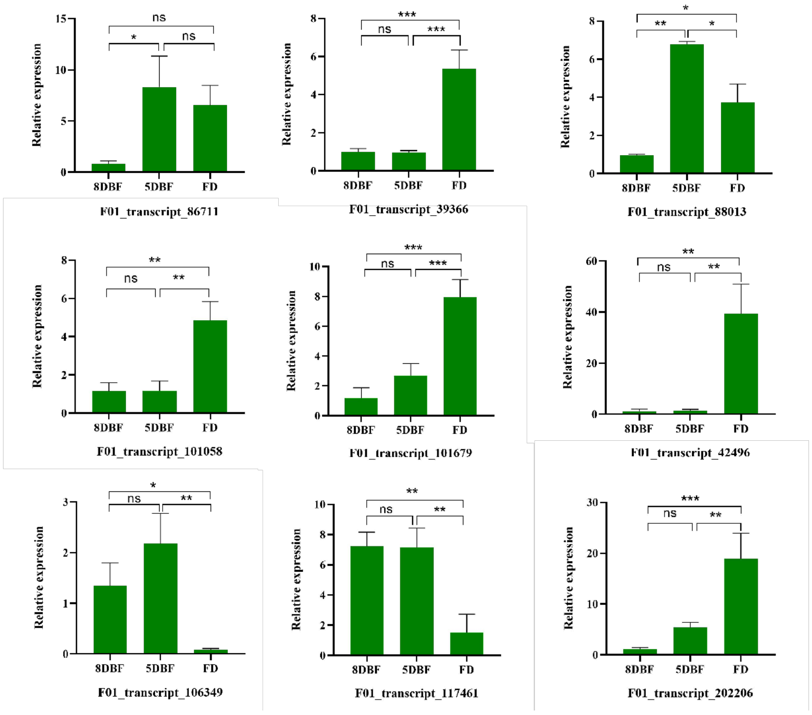

Supplementary Figure S3 Validation of plant gedifferentially expressed genesnes by qRT-PCR
**P*<0.05, ***P*<0.01
